# Circadian Genes Are Expressed during Early Development in *Xenopus laevis*

**DOI:** 10.1371/journal.pone.0002749

**Published:** 2008-07-23

**Authors:** Kristen L. Curran, Silvia LaRue, Brittany Bronson, Jessica Solis, Aaron Trow, Nicole Sarver, Haisun Zhu

**Affiliations:** 1 Department of Biological Sciences, University of Wisconsin-Whitewater, Whitewater, Wisconsin, United States of America; 2 Department of Biology, University of Virginia, Charlottesville, Virginia, United States of America; Katholieke Universiteit Leuven, Belgium

## Abstract

**Background:**

Circadian oscillators are endogenous time-keeping mechanisms that drive twenty four hour rhythmic changes in gene expression, metabolism, hormone levels, and physical activity. We have examined the developmental expression of genes known to regulate circadian rhythms in order to better understand the ontogeny of the circadian clock in a vertebrate.

**Methodology/Principal Findings:**

In this study, genes known to function together in part of the core circadian oscillator mechanism (*xPeriod1*, *xPeriod2*, and *xBmal1*) as well as a rhythmic, clock-controlled gene (*xNocturnin*) were analyzed using in situ hybridization in embryos from neurula to late tailbud stages. Each transcript was present in the developing nervous system in the brain, eye, olfactory pit, otic vesicle and at lower levels in the spinal cord. These genes were also expressed in the developing somites and heart, but at different developmental times in peripheral tissues (pronephros, cement gland, and posterior mesoderm). No difference was observed in transcript levels or localization when similarly staged embryos maintained in cyclic light were compared at two times of day (dawn and dusk) by in situ hybridization. Quantitation of *xBmal1* expression in embryonic eyes was also performed using qRT-PCR. Eyes were isolated at dawn, midday, dusk, and midnight (cylic light). No difference in expression level between time-points was found in stage 31 eyes (p = 0.176) but stage 40 eyes showed significantly increased levels of *xBmal1* expression at midnight (RQ = 1.98+/−0.094) when compared to dawn (RQ = 1+/−0.133; p = 0.0004).

**Conclusions/Significance:**

We hypothesize that when circadian genes are not co-expressed in the same tissue during development that it may indicate pleiotropic functions of these genes that are separate from the timing of circadian rhythm. Our results show that all circadian genes analyzed thus far are present during early brain and eye development, but rhythmic gene expression in the eye is not observed until after stage 31 of development.

## Introduction

Many types of physiology and behavior are controlled by circadian clocks in vertebrates. These endogenous timing mechanisms allow synchronization of important physiological events with the outside world. The circadian clock is composed of a set of interlocking transcription/translation feedback loops which are well conserved among animals [1]–[2]. The central “core” negative feedback loop is essential for clock function and is composed of a set of clock genes, *Period* (*Per*) and *Cryptochrome* (*Cry*), which are transcriptionally activated by a heterodimeric transcription factor composed of CLOCK and BMAL1. As *Per* and *Cry* levels increase, they result in the accumulation of PER and CRY proteins which form complexes with each other and eventually translocate into the nucleus where they inhibit the activity of CLOCK/BMAL1 and repress the transcription of their own genes. Eventually, the repressive complex is degraded and the repression is relieved and the cycle can begin again. This cycle takes approximately 24 hours and defines the circadian day. This core oscillator then influences the expression of output genes like *xNocturnin* [3]–[4] which affect the different physiological and behavioral changes associated with circadian rhythms. For example, *Nocturnin* in mice has been found to influence both lipid and carbohydrate metabolism [5].

Despite the new advances in the understanding of circadian clocks in adult organisms, the ontogeny of circadian rhythms has been less well studied. In this manuscript, we examine the developmental patterns of circadian clock gene expression in *Xenopus laevis* embryos. Work by Green et al. [6] shows that a fully functional circadian system is present in the pineal gland twenty-nine hours post fertilization (hpf; stage 26) and seventy-six hpf (stage 41) in the retina. Although these findings demonstrate that clocks are present and functional by these stages, it is possible that they are present earlier in development. Consistent with this idea, a component of the central oscillator (*Clock*) is expressed at very early gastrula stages (stage 10–11) in the Spemann's organizer [7].

In order to begin analysis of how the circadian oscillator and its outputs are assembled during early development in a vertebrate we examined the early expression pattern of four *Xenopus* clock genes, *xPeriod 1(xPer1)*, *xPeriod 2 (xPer2)*, *xBmal1*, and x*Nocturnin*. *xPer1, xPer2*, and *xBmal1* are components of the central oscillator, while *xNocturnin* is controlled by the clock. We also began to analyze when the circadian oscillator became functional in the developing eye. Our results show that these genes are expressed early in development and may have functions that are not related to circadian rhythm. They further suggest that rhythmic expression of these genes may not occur until the specific organ or tissue is fully differentiated.

## Results

**Figure 1:**
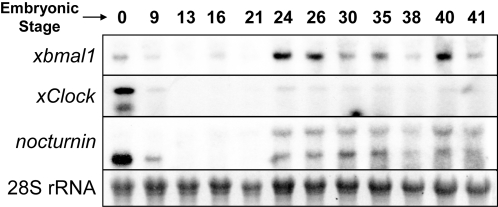
*xClock*, *xBmal1* and *xNocturnin* are expressed as maternal messages before zygotic expression is observed at stage 24. Shown are northern blots performed on RNA isolated from whole embryos at the indicated stages in 12L:12D cycle. Three micrograms of total RNA was loaded into each lane. 28S RNA was used as a loading control.

We began by characterizing the general developmental expression of circadian genes in cyclic light using Northern blot analysis (Figure 1). We found that central oscillator genes like *xClock* and *xBmal1*, as well as an output gene (*xNocturnin)* were expressed at high levels in one celled embryos which indicated that they were maternally expressed. As development continued, the maternal mRNA of these genes gradually diminished. At early tailbud stages (stage 21–24; 22–26 hpf), the mRNA levels of these genes increased, suggesting the activation of zygotic gene expression. The onset of zygotic gene expression was the same in constant conditions (light or dark; data not shown).

We next used whole mount in situ hybridization to analyze the expression patterns of *xPer1*, *xPer2*, *xBmal1*, and *xNocturnin* from early neurula stages (stage 14) to late tailbud stages (stage 39/40) of development. In all of the in situ experiments outlined below, the developmental expression of each gene was characterized at both dawn (lights on; zeitgeber time (ZT) 0) and dusk (lights off; ZT12). Eggs were fertilized at different times of the day and night and cultured at a constant temperature. We were then able to obtain a specific stage of development at a specific time during the day or night. Embryos that were the same relative stage, but were taken at dusk vs. dawn, were analyzed in parallel by in situ hybridization. No obvious time of day difference in the expression pattern or levels of expression was observed (data not shown).

### Characterization of *xPer1* and *xPer2* expression

#### Neurula and neural tube stages

**Figure 2:**
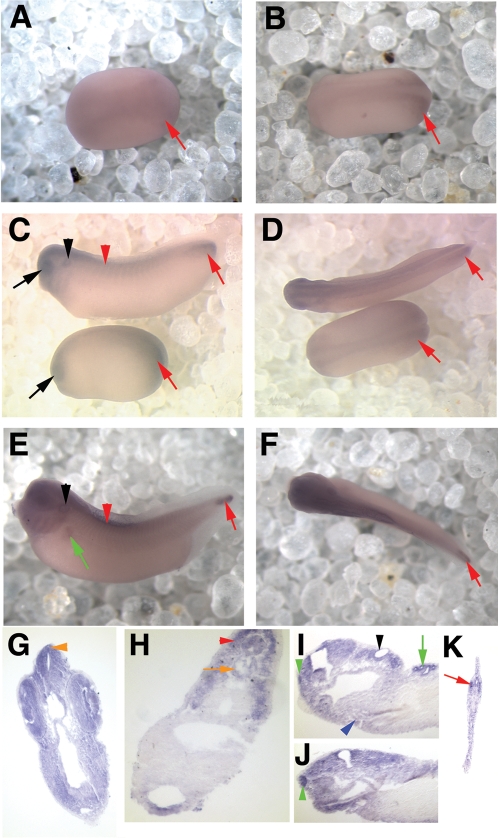
*xPer1* is expressed from neural plate to late tailbud stages. Shown are in situ hybridization results depicting expression of *xPer1* mRNA. Panels A, B, D, and F show dorsal views of the embryos and panels C and E show side views of the embryos. All whole mount embryos (as well as panel I) are oriented with the anterior facing left. Dorsal is toward the top in all images. A and B show neural plate staining at stage 15/16 and stage 18, respectively. Red arrows denote posterior mesoderm staining (A–F). C and D show a neural tube stage embryo on the bottom (stage 22) and an early tailbud stage embryo on top (stage 33). The black arrow denotes eye expression and the red arrowhead shows somite staining. C and D also show *xPer1* expression in the CNS and posterior mesoderm (red arrow), as well as the otic vesicle (C, black arrowhead). Panels E and F depict *xPer1* expression in the CNS, somites (red arrowhead), otic vesicle (black arrowhead), pronephric tubules (green arrow) and posterior mesoderm (red arrow) in a late tailbud stage embryo. Panels G–J show sections of late tailbud embryos (G–H and J are transverse sections and I is a sagittal section). G shows expression in the neural tube, retina, lens, and pineal gland (orange arrowhead). H shows expression in the notochord (orange arrow) and the somites (red arrowhead). In panel I, the olfactory pit (green arrowhead), otic vesicle (black arrowhead), heart (blue arrowhead) and pronephros (green arrow) were stained. Panel J shows olfactory pit staining (green arrowhead). Panel K shows posterior mesoderm staining in the tail tip (red arrowhead).

**Figure 3:**
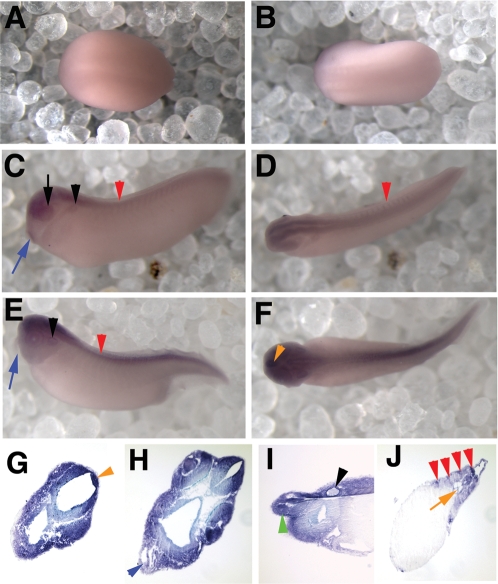
*xPer2* is expressed from neural plate to late tailbud stages. Shown are in situ hybridization results depicting expression of *xPer2* mRNA. Panels A, B, D, and F show a dorsal view of each embryo. Panels C and E show side views. All embryos are oriented with the anterior to the left. G–I show transverse sections and J shows a sagittal section of late tailbud stage embryos. Sections shown in G, H, and J are oriented with the dorsal side at the top right of the panel. Neural plate staining is shown in panel A (stage 16) and B (stage 18). C and D depict early tailbud embryos with continued expression in the CNS as well as in the eye (black arrow), otic vesicle (black arrowhead), cement gland (blue arrow) and somites (red arrowheads). In late tailbud embryos (E and F), *xPer2* is expressed in the otic vesicle (E,I, black arrowhead), pineal (F,G, orange arrowhead), brain, retina, lens (G), and olfactory pit (I, green arrowhead), although cement gland staining was lost (E, blue arrow). *xPer2* was also present at low levels in the heart (H, blue arrowhead) and notochord (J, orange arrow). J also shows somite staining (red arrowheads).

Expression of *xPer1* and *2* was first seen during early neural plate stages, soon after gastrulation was completed. At stage 14 of development (approximately 16 hpf; [8]) no *xPer1* or *xPer2* expression was detected. One hour later (stage 15; 17 hpf) light staining of the neural plate was detectable for both genes. A dorsal view of neural plate staining in Stage 16 (18 hpf) embryos is shown in Figure 2A (*xPer1*) and Figure 3A (*xPer2*). The level of expression in the neural tissue increased as the embryos aged as shown in Figure 2B and 3B, which display expression of *xPer1* and *xPer2*, respectively, in stage 18 embryos (20 hpf; neural groove stage). During early neural tube stages we first observed both *xPer1* and *2* in the developing eye (*xPer1* shown in Figure 2C, lower embryo, black arrow; stage 22 (24 hpf)).

During these early stages of development one obvious difference was seen between *xPer1* and *xPer2* expression. A high level of *xPer1* was expressed in the posterior mesoderm of the neurula and neural tube stage embryos (Figure 2A and B, red arrows). No *xPer2* expression was observed in the posterior mesoderm. *xPer1* continued to be expressed at high levels in the tip of the tail well into late tadpole stages (Figure 2 E, red arrow).

#### Tailbud stages

During early tailbud stages *xPer1* and *2* are present and similarly expressed in the developing central nervous system (CNS), eye (black arrow), otic vesicle (black arrowhead), branchial arches and in the somites (red arrowhead) (Figure 2 C–D; Figure 3 C–D). *xPer1* and *xPer2* are first detectable in the somites around stage 24/25 (about 26 hpf). They are expressed at low levels in the somites well into tadpole stages (Figure 2 and 3 C–F, red arrowheads).

*xPer1* and *xPer2* have markedly different expression patterns in the cement gland. *xPer2* is present in the developing cement gland during early tailbud stages, but is lost during late tailbud stages (compare Figure 3C and E, respectively, blue arrows), while *xPer1* is not detectable in the cement gland at any of the developmental stages examined.

Expression of these two genes continues in the CNS, eye, otic vesicle, pineal, and somites of late tailbud stage embryos (Figure 2E,G,H, *xPer1*; Figure 3E–J, *xPer2*). *xPer1* expression was first detected in the pronephric tubules at late tailbud stages (stage 39; 56 hpf) (Figure 2 E,I, green arrow). *xPer2* was not observed in the pronephric tubules.

We confirmed staining of specific structures during late tailbud stages by sectioning embryos that had first been analyzed by whole mount in situ hybridization. *xPer1* and *2* were present in the pineal gland, visible in whole mount for *xPer2* (Figure 3F, orange arrowhead) and in sections (Figure 2 and 3 G; orange arrowhead). We also observed expression in the brain, retina and lens for both genes (Figure 2G (*xPer1*) and Figure 3H (*xPer2*)). Both genes were present in the neural tube, notochord (orange arrow) and somites (red arrowhead) although stripes of *xPer-2* expression (Figure 3J, red arrowheads) were seen that were not apparent in the somites stained for *xPer-1* (Figure 2H). Low levels of *xPer-1* and *2* were present in the heart (Figure 2I, Figure 3H (blue arrowhead)). Expression of both genes was seen in the olfactory pit (green arrowhead) and otic vesicle (black arrowhead) (Figure 2I,J and Figure 3I). Lastly, we confirmed that the expression of *xPer1* was present in the posterior mesoderm (Figure 2K, red arrowhead).

### Characterization of *xBmal1* expression

#### Neurula and neural tube stages

**Figure 4:**
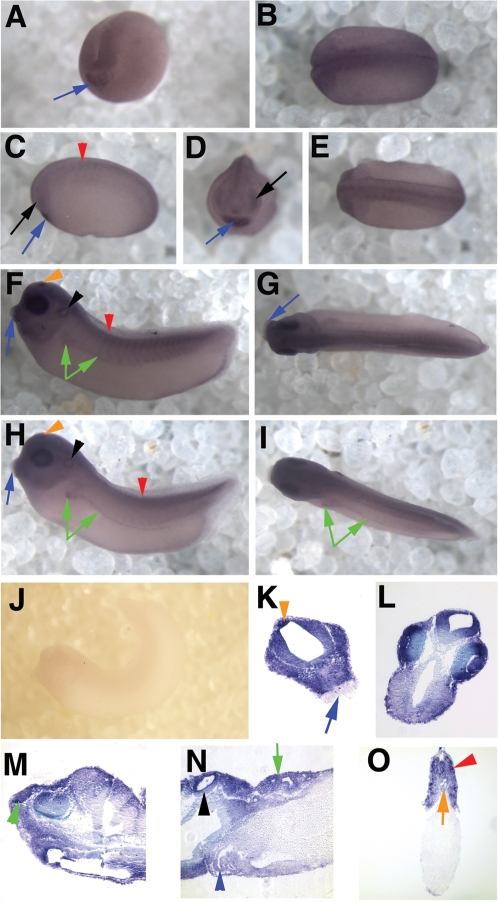
*xBmal1* is expressed from neural plate to late tailbud stages. Shown are in situ hybridization results depicting expression of *xBmal1* mRNA. All embryos are oriented with the anterior to the left in all panels except A and D. Panel A shows the embryo from the anterior, but slightly angled to one side. D shows an anterior view. Panels C, F, H and J show side views of the embryos. Panels B, E G, and I show dorsal views of the embryos. Panels K–M and O show transverse sections and panel N shows a sagittal section of late tailbud embryos. Panel A and B depict a stage 18 embryo with *xBmal1* staining in the neural plate and cement gland (blue arrow). C–E show stage 23 (neural tube stage) embryos with expression in the eye (black arrow), cement gland (blue arrow), and somites (red arrowhead). F–I show early (F–G) and late (H–I) tailbud stages where *xBmal1* is expressed in the eye, pineal (orange arrowhead), otic vesicle (black arrowhead), somites (red arrowhead) and the pronephric tubules and duct (green arrows). Cement gland staining was lost (blue arrow). K and L show expression in the brain, retina/lens, pineal (orange arrowhead), and absence of staining in the cement gland (blue arrow). M–O show expression in the olfactory pit (green arrowhead), otic vesicle (black arrowhead), pronephric tubules (green arrow), heart (blue arrowhead), somites (red arrowhead), and notochord (orange arrow). No expression was seen using a sense probe specific to *xBmal1* (J).

*xBmal1* is first detectable in the neural plate and cement gland (blue arrow) of stage 15 embryos (Figure 4 A shows a stage 18 embryo; 20 hpf). A dorsal view of the same embryo is shown in Figure 4B and depicts expression of *xBmal1* throughout the neural plate. *xBmal1* expression continues to increase in the CNS and head during neural tube stages. This gene is first detectable in the eye around stage 24/25 (Figure 4C and D; black arrows; 26–27 hpf). We faintly detect expression of *xBmal1* in the developing somites during these stages (Figure 4C, red arrowhead). Cement gland staining remains robust (Figure 4C and D; blue arrows). Figures 4B and 4E show a dorsal view from neurula and neural plate stages respectively.

#### Tailbud stages

During early tailbud stages *xBmal1* was detected in the CNS, eyes, pineal (orange arrowhead), otic vesicles (black arrowhead), pronephric tubules and pronephric duct (green arrows) (Figure 4F–G, early tailbud; 4H–I late tailbud). Cement gland staining decreased during early tailbud stages and was absent by late tailbud stages (compare Figure 4F and H, blue arrows). No staining was observed using a sense control for *xBmal1* (Figure 4J).

We again confirmed our observations by sectioning late tailbud embryos that had been analyzed by whole mount in situ hybridization for *xBmal1*. *xBmal1* was found in the pineal gland (orange arrowhead) but was absent in the cement gland (Figure 4K, blue arrow). *Xbmal1* was present in the developing brain, retina, and lens (Figure 4L) as well as the olfactory pit (Figure 4M, green arrowhead), otic vesicle (Figure 4N, black arrowhead) heart (4N, blue arrowhead) and pronephric tubules (4N, green arrow). *Xbmal1* was also present in the notochord (orange arrow) and somites (red arrowhead) (Figure 4O). Specific staining was also observed around the anus/blastopore region at late tailbud stages both in wholemount and section (not shown in figure 4, but present in figure 6E as a wholemount).

### Characterization of *Nocturnin* expression

#### Neurula and neural tube stages

**Figure 5:**
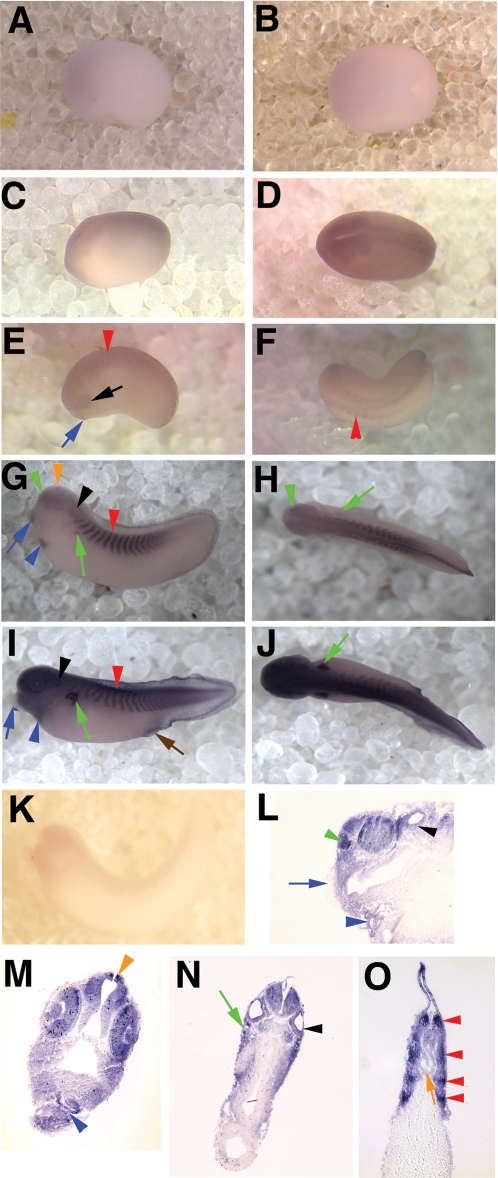
*xNocturnin* is expressed from neural plate to late tailbud stages. Shown are in situ hybridization results depicting expression of *xNocturnin* mRNA. All embryos in this figure are shown with the anterior facing left. Side views of the embryos are depicted in panels A,C,E,G,I, and K and dorsal views in panels B,D,F, H, and J. Low levels of *xNocturnin* were first detected in the neural plate of stage 15/16 embryos A and B. C and D show neural plate staining in a stage 18 embryo. E and F show a neural tube stage embryo (stage 24) with *xNocturnin* expression in the eyes (black arrow), somites (red arrowhead), and cement gland (blue arrow). G and H show early tailbud stage embryos with staining in the otic vesicle (black arrowhead), pronephric tubules (green arrow), heart (blue arrowhead), olfactory pit (green arrowhead), pineal (orange arrowhead), cement gland (blue arrow) and somites (red arrowhead). Late tailbud stages (I and J; stage 39) show similar results but additional staining in the anus/blastopore (brown arrow) and cement gland staining is absent (blue arrow). Sagittal (L) and transverse sections (M–O) of late tailbud embryos confirm *xNocturnin* expression in the brain, retina and lens (M), otic vesicle (N, black arrowhead), olfactory pit (L, green arrow), pronephric tubules (N, green arrow), heart (M, blue arrowhead), notochord (O, orange arrow) and in the somites (O, red arrowheads). *xNocturnin* is absent from the cement gland at late tailbud stages (L, blue arrow). No expression was seen using a sense probe specific to *Nocturnin* (K).

*xNocturnin* was first detectable during early neurula stages. Light staining at stage 15/16 of neural plate stage embryos is shown in Figure 5A (side view) and 5B (dorsal view). By stage 18 (20 hpf), *xNocturnin* was easily observed in the developing CNS but not in the cement gland (Figure 5C and D). During neural tube stages *xNocturnin* was detected in the cement gland (blue arrow) and developing eyes (black arrow), as well as the somites (red arrowhead) (Figure 5 E,F; stage 24).

#### Tailbud stages

During early tailbud stages *xNocturnin* expression was initiated in different organs at slightly different times. Expression of *xNocturnin* in the heart was first observed at stage 27 (Figure 5G, blue arrowhead; 31 hpf). *xNocturnin* was first observed in the pronephric tubules at stage 28 (Figure 5 G–H green arrow; 32 hpf). Expression in the pineal was first observed at stage 29 (Figure 5 G orange arrowhead; 35 hpf). During early tailbud stages *xNocturnin* was always observed in the eyes (Figure 5 G–H), olfactory pit (G–H, green arrowheads), otic placodes (G, black arrowheads), cement gland (G, blue arrow), and somites (G, red arrowhead).

During late tailbud stages, cement gland staining was lost at stage 33 (Figure 5 I and L, blue arrow; 44 hpf). *xNocturnin* was also observed around the anus or blastopore region during tailbud stages (Figure 5 I, brown arrow). At this stage, *xNocturnin* was highly expressed in the head when compared to early tailbud stages. Sections of late tailbud embryos specifically showed expression in the otic placodes (black arrowhead), the brain, retina, and lens (Figure 5M), olfactory pit (Figure 5L, green arrowhead), otic vesicle (Figure 5 L and N, black arrowhead) and pineal (Figure 5M, orange arrowhead). *xNocturnin* expression in the heart (Figure 5L and M, blue arrowhead) and pronephric tubules (Figure 5N,green arrow) was also confirmed in sections. Stripes of staining in the somites (Figure 5O, red arrowheads) were apparent in transverse sections, but not in whole mount or sagittal section. A sense probe specific to *xNocturnin* was negative (Figure 5K).

### Comparison of the spatial and developmental expression of *xPer1*, *xPer2*, *xBmal1*, and *xNocturnin*

#### Differences in spatial expression in the somites

**Figure 6:**
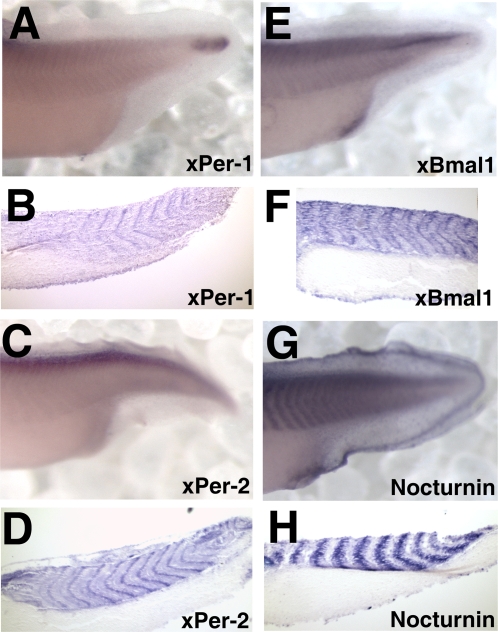
A comparison of somite staining in the posterior of late tailbud embryos (stage 36–38). Shown are in situ hybridization results depicting RNA expression in paired whole mount and sagittal sections of the posterior of embryos stained with *xPer1* (A–B), *xPer2* (C–D), *xBmal1* (E–F), and *Nocturnin* (G–H).

We observed a difference in the spatial expression of *xPer1*, *xPer2*, *xBmal1*, and x*Nocturnin* in the somites (mesoderm). The three central oscillator components (*xPer1*, *xBmal*, and *xPer2)* were expressed at the anterior and posterior margins of each somite. Figure 6 A–F show *xPer1*, *xPer2*, and *xBmal1*, respectively, in both whole mount and sagittal section. In contrast, *xNocturnin* is expressed throughout the somite or between regions where the central oscillator genes were expressed (Figure 6G–H). Also, as mentioned above, transverse sections of *xPer2* and *xNocturnin* expression showed a striping pattern which was not observed in *xPer1* or *xBmal1* stained embryos.

#### Differences in the temporal order of expression of each gene during development

**Figure 7:**
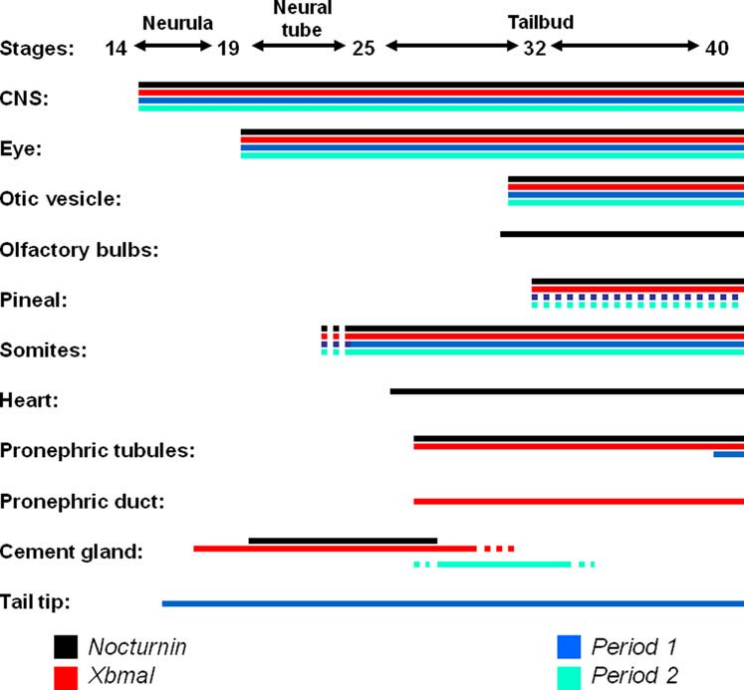
A temporal summary of the expression patterns of *xPer1*, *xPer2*, *xBmal1*, and *Nocturnin*. The approximate stages of development are represented on the horizontal axis of this figure while the particular tissues and organs are listed on the vertical axis. *xPer1* is represented by the blue lines, *xPer2* by the green lines, *xBmal1* by the red lines, and *Nocturnin* by the black lines. Dotted lines indicate times during development when a gene may be present, but was not confirmed through sectioning or additional whole mount in situ analysis.

Interestingly, each gene analyzed had a unique developmental expression pattern outside of the CNS and in sensory structures. All four genes were present in the heart, but *Nocturnin* was the most strongly expressed. *Nocturnin*, *xBmal1*, and *xPer1* were all present in the pronephric tubules, but *xPer1* was only detectable at late tailbud stages. Also, *xBmal1* was the only gene found to be expressed in the pronephric duct. *Nocturnin*, *xBmal1*, and *xPer2* were found to be expressed in the cement gland but for different periods of time. *xPer1* was not detectably expressed in the cement gland. Lastly, *xPer1* was found to be expressed at high levels in the posterior mesoderm, in contrast to the other three genes. These observations are summarized in Figure 7.

### Rhythmic expression of *xBmal1* in the eye in cyclic light

**Figure 8:**
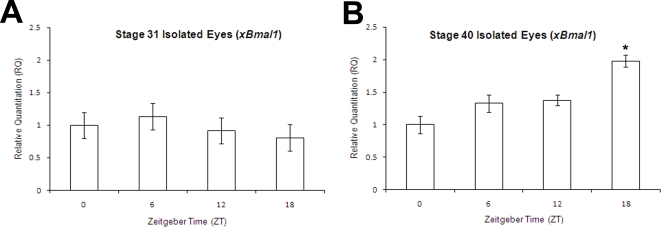
Isolated eyes show rhythmic expression of *xBmal1* at stage 40 but not at stage 31. Eyes were dissected from embryos maintained in a 12L:12D cycle at different stages of development and different circadian times (ZT 0 (dawn), ZT6 (mid-day), ZT12 (dusk),and ZT18 (midnight)). The eyes were analyzed by qRT-PCR. The relative quantitation (RQ) of *xBmal1* for each sample was calculated with respect to EF1α. No difference in the levels of *xBmal1* expression was observed in stage 31 embryonic eyes at any time of day tested (ANOVA; df3, F = 1.77, p = 0.176; arrhythmic). A significant difference in *xBmal1* expression was observed when all ZTs were analyzed in stage 40 embryonic eyes (ANOVA; df3, F12.23, p = 0.00009). The asterisk shows that the level of *xBmal1* expression at ZT18 was significantly different from ZT0 (ANOVA, df1, F = 27.82, p = 0.0004). Bars in each graph denote standard error.

An analysis was done to assess when circadian rhythm might begin in the developing eye. The eye was chosen because it is easily dissected from the embryo and is known to have a circadian rhythm at stage 41 of development, but not at stage 26 [6]. During the experiment the parents and embryos were maintained in a 12 hour light:12 hour dark (12L:12D) cycle. Eyes were dissected from stage 31 and stage 40 embryos at dawn (ZT0), midday (ZT6), dusk (ZT12), and midnight (ZT18) and analyzed for expression of *xBmal1* and EF1α (endogenous control) using quantitative real time PCR (qRT-PCR). *xBmal1* was used as a marker of endogenous regulation of rhythmic gene expression because it does not directly respond to light [9]. The stage 31 embryonic eyes were arrhythmic, with no significant difference in the levels of expression at four different time points (ANOVA; df3, F = 1.77, p = 0.176; Figure 8A). Stage 40 embryonic eyes did show time of day specific differences in expression of *xBmal1* (ANOVA; df3, F = 12.23,p = .00009; Figure 8B). Single factor ANOVA was used to compare the expression of *xBmal1* at ZT6, ZT12, and ZT18 with ZT0. Only ZT18 was significantly different from ZT0 (ANOVA; df1, F = 27.82, p = 0.00036, asterisk in Figure 8B). This result was repeated in a second trial and the same pattern was observed in the second trial.

Whole embryos representing the age and ZT of the isolated eyes in Figure 8 were fixed and analyzed for *xBmal1* expression by in situ hybridization. No significant difference in expression pattern or level of expression in the eye or other tissues was seen in stage 31 and stage 40 embryos when ZT 0, 6, 12, and 18 were compared (data not shown).

## Discussion

Our initial northern blot analyses showed that the circadian clock genes *xClock*, *xBmal1* and the output gene *xNocturnin* are maternally expressed during early embryonic stages. Zygotic expression of *xPer1*, *xPer2*, *xBmal1*, and *xNocturnin* genes was first detected by in situ hybridization during early neurogenesis (neural plate stages, stage 15) and these genes were expressed widely in the developing central and peripheral nervous system, including the brain and spinal cord, the pineal gland, otic vesicle, olfactory pit and in the eyes. Our higher resolution analysis for rhythmic expression of *xBmal1* suggests that the circadian oscillator must become fully mature in the eye between stage 31 and 40 of development.

### Developmental expression of circadian genes

By in situ, we see localized expression of four circadian genes (*xBmal1*, *xPer1*, *xPer2* and *xNocturnin*) in the neural plate just after the beginning of neurulation (stage 15; 17 hpf). This result was expected since low levels of zygotic expression of *xBmal1* and *Nocturnin*, as well as another central oscillator gene (*xClock*), were observed during neurula and neural tube stages in a northern blot (Figure 1). Ziv and Gothilf [10] noted that ubiquitous expression of *zper2* mRNA is first detected during blastula stages and during the six somite stage is localized to the neural plate in zebrafish. We have not analyzed blastula stages by *in situ*, but northern blot analysis indicated that the maternal message of two central oscillator components (*xClock* and *xBmal1*) is present during blastula stages (stage 9; 7–9 hpf), but decreased and was replaced by zygotic transcription later in development.

As development progresses, we see stage specific differences in the expression of *xPer1*, *xPer2*, *xBmal1* and *xNocturnin* in organs and tissues of the developing frog embryo. Each gene analyzed had a unique developmental expression pattern outside of the CNS. Figure 7 provides a summary of the temporal developmental expression of these four genes that was detectable by whole mount in situ hybridization. Since we compared the expression of each gene at each stage of development at both dawn and dusk, we are convinced that the changes in expression that we see are due to developmental changes and not differences in expression due to the time of day. Also, we performed sense controls for both *xBmal1* and *xNocturnin* (Figures 4J and 5K, respectively). In both cases no specific staining was observed and background staining was minimal. The results from the sense controls are consistent with our interpretation that the definable differences in spatial and developmental expression of each of the four genes analyzed were specific for each gene. The differences in expression pattern observed for each gene may point to unique non-circadian roles for these genes in the development of the pronephros, heart, and other sensory structures. In addition, these genes were in some cases expressed in each structure at distinct developmental times. For example, *Nocturnin* and *xBmal1* were present in the cement gland at the same time, but *xPer2* was expressed later in development after these mRNAs were no longer detected (Figure 7).

One general question that can be addressed given our findings is whether there is a need for a functional circadian oscillator in developing organs and tissues or whether these genes are playing some other role at this time. It is known that peripheral tissues in adult organisms can have circadian oscillators [11]. These peripheral clocks are coordinated by the “master” clock in the SCN of mammals and are thought to regulate rhythmic changes within their respective tissues [12]. For example, in the adult mammalian heart, gene expression, heart rate, and systolic blood pressure are all under the control of the local heart circadian oscillator [2], [13]. Therefore, it may be that the expression of these clock genes in these tissues in early development means that the peripheral clocks are established soon after tissues or organs develop, similar to what is seen in the *Xenopus* and zebrafish pineal [6], [14]. However, when the full cohort of circadian oscillator genes is not coexpressed in a tissue it is likely that they are performing some other function.

Several examples exist that suggest that clock-related genes may play other roles during development. For example, in *Xenopus*, Morgan [15] reported that *Pax-6* expression was activated by *xClock*. Because *Pax-6* is known to be a master control gene in eye development, these data suggest that the presence of these genes in the eye may not only provide circadian function, but may also directly affect retinal development. In another interesting example, it has been suggested that homologs of circadian genes in *C. elegans* do not play a role in circadian rhythmicity, but are instead involved in developmental timing [16]–[18]. Circadian genes like *Tim-1* and *Kin-20* (homologs of *timeless* and *doubletime*, respectively, in *Drosophila*) affect developmental timing genes like *lin-42* in *C. elegans* [17]. *Lin-42* is a homolog of the *Drosophila* and mouse *Period* gene (34% and 28% identity) [17]. It may be that circadian genes in vertebrates also have pleiotropic functions that are important for development that are separate from the timing of circadian rhythms.

No difference in expression level or pattern was observed when comparing the expression of *xPer1*, *xPer2*, *xBmal1* and *xNocturnin* circadian genes at ZT0 or ZT12 as well as in an experiment analyzing whole embryos at stage 31 and stage 40 at ZT 0, 6, 12, and 18 using in situ hybridization (data not shown). When *zPer2* expression was measured in zebrafish, circadian changes were detected after 24 hours of development in the pineal using in situ analysis methods similar to ours [14]. We were unable to detect rhythmic expression of *xBmal1* by in situ hybridization in eyes that have been shown to be rhythmic by qRT-PCR (Figure 8B). This suggests that, at least in *Xenopus*, it is difficult to assay quantitatively for rhythmic changes in gene expression by in situ hybridization.

The somitic expression of *xPer1*, *xPer2*, *xBmal1*, and *xNocturnin* during development is interesting and suggests that circadian genes may be involved in the formation, maintenance, and/or timing of somitogenesis. *Nocturnin*, a deadenylase [4], was strongly expressed throughout each somite. *xPer1, xPer*2, and *xBmal1* were expressed at the anterior and posterior margins of each somite (Figure 6). It is possible that building a somite or maintaining the borders between developing somites requires regulation of gene expression by circadian genes. Another possibility is that these genes play a role in the timing of somitogenesis.

It is interesting to postulate that genes that are known to play a role in circadian timing may also influence the cell autonomous somitogenesis timer. The periodic formation of somites during development is controlled by members of the Notch signal transduction cascade [19]. However, *xPer1, xPer2, xBmal1,* and *xNocturnin* are not obviously expressed in the presomitic mesoderm like many other somitogenesis timing candidates in chick (*hairy 1*, *hairy 2* and *lunatic fringe;* [20]). There are two components that may be common to both the segmentation clock in somites and the circadian clock (GSK-3 and Casein kinase IIα; [21]). It is possible that circadian genes act as a counting mechanism, play a role in maintaining the timing mechanism for somitogenesis, or they may play a unique role during somitogenesis that we have yet to elucidate.

### Onset of rhythmic gene expression in the embryonic eye

In this paper we show that we can detect rhythmic expression of *xBmal1* in stage 40 eyes but not in stage 31 eyes using qRT-PCR (Figure 8). For these experiments we analyzed one tissue (the eye) instead of the whole embryo because circadian genes are known to come on at different developmental times and can become rhythmic or not at different times during development which could influence the detection of a definable rhythm in whole embryos [22]–[23]. The eye dissection for these experiments was, by necessity, done with the lights on, therefore we needed to analyze a circadian gene that was not actually activated by light. Immediate early transcription of *xPer1* and *xPer2* in response to light and *xNocturnin* in response to serum shock and TPA has been reported in mammals [24]–[25]. *zPer-2* has also been shown to be light sensitive [14]. *xBmal1* expression is not directly influenced by light making it the best candidate to assay [9].

Our results suggest that circadian genes are first expressed constitutively in the developing eye, and as the eye matures gene expression then becomes rhythmic. Our initial experiments analyzed whole embryos for circadian gene expression of *xBmal1* and other genes at dawn (ZT0) and dusk (ZT12) using in situ hybridization. We chose to sample these two time points given the difficulty of this type of experiment and also based on the circadian profile of the target genes, ie., morning phased or evening phased. Unfortunately, this approach may fail to detect rhythmic expression if peaks and troughs of expression are significantly shifted from the two times of day analyzed. Better discrimination of rhythmic gene expression can be achieved by analyzing embryos or tissues at additional times of the day and night (ZT0, 6, 12, and 18) and using a more sensitive analysis tool (qRT-PCR). *xBmal1* was first detectable in the developing eye at stage 24/25 (26–27 hpf) by in situ hybridization but is not rhythmically expressed in the eye until sometime after stage 31 (37 hpf). Other tissues in the developing embryo may also display this same paradigm. In the future, we intend to analyze peripheral tissues, such as heart, otic vesicle, and pronephros, using qRT-PCR to determine when rhythmic expression of circadian genes begins during development.

Determining the precise onset of rhythmic circadian gene expression in the eye is also a subject of ongoing research in our lab. The results shown in Figure 8 indicate that the onset of rhythmic expression of a central oscillator gene (*xBmal1*) occurs between stages 31 and 40 of eye development in *Xenopus laevis*. Previous studies in both *Xenopus* and zebrafish also indicate that the maturation of circadian rhythm in the eye occurs much later in development than the pineal gland [6], [14], [26]. We are interested in studying the maturation of the circadian oscillator and its subsequent influence on clock controlled genes in the embryonic eye of *Xenopus laevis*. Eye development and maturation in *Xenopus* is a slow process (approximately 60 hours in *Xenopus,* calculated from data in [8]) and may allow us to more precisely identify how and in what cell types a circadian system (oscillator controlling clock controlled genes) is assembled in a specific tissue during development.

## Materials and Methods

### Obtaining embryos

An albino strain of *Xenopus laevis* (NASCO) was used for the in situ hybridization experiments. Pigmented embryos were used for other analyses. Eggs were collected from females injected with 800 units of human chorionic gonadotropin (Westminster Veterinary Supply) and fertilized with macerated testis. Embryos were then maintained in a low ionic strength salt solution, 1/3X Modified Barth's Solution (MBS; [27]). The developmental expression of *xPer1*, *xPer2*, *xBmal1*, and *xNocturnin* were analyzed by in situ hybridization between stage 14 (early neurula) and stage 39/40 (late tailbud) of development. All embryonic stages were determined according to Nieuwkoop and Faber [8]. The females were maintained in a 12L: 12D cycle for two weeks before eggs were collected. The eggs/embryos were maintained in this same cyclic light regime. This was done so that if there was some maternal effect on the circadian rhythm of the eggs and embryo, it would not be compromised by using a different light/dark cycle. In order to obtain embryos at a specific stage of development and at two different zeitgeber times (ZT) we induced egg laying by the females at various times during the day and night. We then fertilized the eggs at times that should yield the stage of development we wanted to obtain at ZT0 (dawn) or ZT12 (dusk). Embryos were maintained at 22°C.

A higher resolution analysis of the expression of *xBmal1* in the developing eye and embryo was also performed. Embryos were obtained from parents that were maintained in a 12L:12D cycle at 19°C. The embryos were cultured under the same light regime and temperature as the adults. Egg laying was again induced at various times of the day and night to obtain stage 31 or 40 embryos at ZT0 (dawn), ZT6 (midday), ZT12 (dusk), and ZT18 (midnight). Two eyes were dissected from each embryo using a Leica S4E stereoscope under normal fluorescent light (25 minutes to dissect and freeze eyes). Duplicate samples at each stage and ZT were collected by placing eyes isolated from an individual embryo in two separate tubes (ten eyes per tube representing ten individual embryos). The embryos were then frozen on dry ice and stored at −80°C for further analysis of *xBmal1* expression by quantitative Real Time PCR. Also, stage 31 and 40 embryos were fixed for in situ hybridization at ZT 0, 6, 12, and 18.

### Northern Blot Analysis

Embryos were maintained at 14°C in 12L: 12D cycle. Twenty embryos were removed every 12 hours over 6 days, the developmental stage of the embryos were recorded by comparing to a standard staging chart [8], and then the embryos were frozen on dry ice. Total RNA was isolated from frozen embryos using TRIZOL extraction agent (Life Technology, Gaithersburg, MD) according to manufacturer's instructions. Northern blot analysis was carried out as previously described by Zhu et al. [28] using riboprobes for *xClock*, *xBmal1*, and *xNocturnin*.

**In situ hybridization** was carried out according to the procedure of Harland [29] as modified by Doniach and Musci [30]. During these studies the levels of gene expression were compared in embryos of different ages that were processed in the same *in situ* experiment (i.e. the enzymatic color reaction was done for the same length of time for each data point), therefore the relative levels of expression for each gene at different stages of development were qualitatively comparable. Sense controls were performed on albino *Xenopus laevis* embryos for *xNocturnin* and *xBmal1* only. Also, the in situ hybridization for the experiments reported in Figure 7, were performed on pigmented embryos. The embryos were bleached after the *in situ* procedure was finished [31].

### Histological Analysis

Embryos that had previously been stained using whole mount in situ hybridization were subsequently fixed for 1 hour in MEMFA. Additional fixation overnight in Bouin's fixative was followed by washes with 70% methanol to remove the picric acid [32]. The embryos were then paraffin embedded and sectioned (10 µm) using standard methods [31].

### Photography

Most whole mount pictures were photographed on a Zeiss Stemi SV11 microscope with Zeiss Axiovision AC camera. The sense controls and pictures in Figure 4A and B were photographed using an Olympus SZX9 stereoscope using an Olympus DP70 digital camera.

### Quantitative Real Time PCR

Total RNA was extracted from eyes obtained from stage 31 and 40 embryos that represented different ZT times (0, 6, 12, and 18) using TRI reagent (Ambion). Each sample was treated with TURBO free DNase (Ambion) to remove genomic DNA. First strand cDNA synthesis using random primers hexamers was performed using a High Capacity cDNA Archive kit (Applied Biosystems). Each RNA sample was also prepared without reverse transcriptase as a negative control (RT-). The cDNA was then amplified using a POWER SYBR Green Master Mix (Applied Biosystems; 25 µl reactions) in an ABI 7300 Series Real Time PCR machine as per the manufacturer's specifications. Target primers: *Xbmal1* forward (TACCTTGGCCTTTGTGATCC); *Xbmal1* reverse (TGGCCCCTATGTTTTACTGC); Endogenous control primers: EF1α forward (TACCAGTTGGTCGTGTGGAA); EF1α reverse (GTAAGGGCTTCATGGTGCAT). A standard curve (two fold dilution series including at least 6 points) was obtained for each primer set and the efficiencies of each primer were calculated using qBase 1.3.5 (2006, developed by Jan Hellemans and Jo Vandesompele at the Center for Medical Genetics, Ghent University Hospital) and used for calculation of the relative quantitation of each sample. Six replicates were done for each sample and the relative quantitation (RQ) and standard error were calculated using qBase 1.3.5 (2006). The ZT0 sample was always used as the calibrator. The RQs for samples of similar age were compared at different times of the day (ZT0, 6, 12, and 18) using single factor ANOVA (Microsoft Office Excel™). Duplicates of each sample were analyzed with similar results.

## References

[1] Bell-Pedersen D, Cassone VM, Earnest DJ, Golden SS, Hardin PE (2005). Circadian rhythms form multiple oscillators: lessons from diverse organisms.. Nature Genetics.

[2] Lowrey PL, Takahashi JS (2004). Mammalian Circadian Biology: Elucidating Genome-Wide levels of Temporal Organization.. Annu. Rev. Genomics Hum. Genet..

[3] Green CB, Besharse JC (1996). Identification of a novel vertebrate circadian clock regulated gene encoding the protein nocturnin.. Proc. Natl. Acad. Sci. U S A.

[4] Baggs J, Green CB (2003). Nocturnin, a deadenylase in *Xenopus laevis* retina. A mechanism for posttranscriptional control of circadian-related mRNA.. Curr Biol.

[5] Green CB, Douris N, Kojima S, Strayer CA, Fogerty J (2007). Loss of Nocturnin, a circadian deadenylase, confers resistance to hepatic steatosis and diet-induced obesity.. Proc Natl Acad Sci U S A.

[6] Green CB, Lian M-Y, Steenard BM, Besharse JC (1999). Ontogeny of circadian and light regulation of melatonin release in *Xenopus laevis* embryos.. Dev Brain Res.

[7] Green CB, Durston AJ, Morgan R (2001). The circadian gene clock is restricted to the anterior neural plate early in development and is regulated by the neural inducer noggin and the transcription factor Otx2.. Mech Dev.

[8] Nieuwkoop PD, Faber J (1994). Normal Table of Xenopus laevis (Daudin)..

[9] Preitner N, Damiola F, Lopez-Molina L, Zakany J, Duboule D (2002). The orphan receptor REV-ERB*α* controls circadian transcription within the positive limb of the mammalian circadian oscillator.. Cell.

[10] Ziv L, Gothilf Y (2006). Circadian time-keeping during early stages of development.. Proc Natl Acad Sci U S A.

[11] Reppert SM, Weaver DR (2002). Coordination of circadian timing in mammals.. Nature.

[12] Yoo SH, Yamazaki S, Lowrey PL, Shimomura K, Ko CH (2004). PERIOD2::LUCIFERASE real-time reporting of circadian dynamics reveals persistent circadian oscillations in mouse peripheral tissues.. Proc Natl Acad Sci U S A.

[13] Young ME (2006). The circadian clock within the heart: potential influence on myocardial gene expression, metabolism, and function.. AJP-Heart.

[14] Ziv L, Levkovitz S, Toyama R, Falcon J, Gothilf Y (2005). Functional development of the zebrafish pineal gland: Light-induced expression of *Period 2* is required for onset of the circadian clock.. J Neuroendo.

[15] Morgan R (2002). The circadian gene Clock is required for the correct early expression of the head specific gene Otx2.. Int J Dev Biol.

[16] Jeon M, Gardner HF, Miller EA, Deshler J, Rougvie AE (1999). Similarity of the C. elegans developmental timing protein LIN-42 to circadian rhythm proteins.. Science.

[17] Banerjee D, Kwok A, Lin SY, Slack FJ (2005). Developmental timing in C. elegans is regulated by kin-20 and tim-1, homologs of core circadian clock genes.. Dev Cell.

[18] Hasegawa K, Saigusa T, Tamai Y (2005). *Caenorhabditis elegans* opens up new insights into circadian clock mechanisms.. Chronobio Int.

[19] Freitas C, Rodrigues S, Saude L, Palmeirim I (2005). Running after the clock.. Int J Dev Biol.

[20] Davis RL, Turner DL (2001). Vertebrate hairy and enhancer of split related proteins: Transcriptional repressors regulating cellular differentiation and embryonic patterning.. Oncogene.

[21] Rida PC, Le Minh N, Jiang YJ (2004). A notch feeling of somite segmentation and beyond.. Dev Biol.

[22] Kaneko M, Cahill GM (2005). Light-dependent development of circadian gene expression in transgenic zebrafish.. http://biology.plosjournals.org/perlserv/?request=get-document&doi=10.1371/journal.pbio.0030034.

[23] Vallone D, Lahiri K, Dickmeis T, Foulkes NS (2007). Start the clock! Circadian rhythms and development.. Dev Dyn.

[24] Travnickova-Bendova Z, Cermakian N, Reppert SM, Sassone-Corsi P (2002). Bimodal regulation of mPeriod promoters by CREB-dependent signaling and CLOCK/BMAL1 activity.. Proc Natl Acad Sci U S A.

[25] Gabarino-Pico E, Niu S, Rollag MD, Strayer CA, Besharse JC (2007). Immediate early response of the circadian polyA ribonuclease nocturnin to two extracellular stimuli.. RNA.

[26] Kazimi N, Cahill GM (1999). Development of a circadian melatonin rhythm in embryonic zebrafish.. Brain Res. Dev Brain Res..

[27] Peng HB, Kay BK, Peng HB (1991). Appendix A: Solutions and Protocols.. Methods in Cell Biology, *Xenopus laevis*: Practical uses in cell and molecularbiology, Vol. 36.

[28] Zhu H, LaRue S, Whiteley A, Steeves TDL, Takahashi JS (2000). The *Xenopus Clock* gene is constitutively expressed in retinal photoreceptors.. Mol Brain Res.

[29] Harland RM (1991). In situ hybridization: an improved whole-mount method for Xenopus embryos.. Methods Cell Biol.

[30] Doniach T, Musci TJ (1995). Induction of anteroposterior neural pattern in Xenopus: evidence for a quantitative mechanism.. Mech Dev.

[31] Sive H, Grainger RM, Harland RM (2000). Early Development of *Xenopus laevis*: A Laboratory Manual, Cold Spring Harbor Press, New York..

[32] Sasai Y, Lu B, Piccolo S, De Robertis EM (1996). Endoderm induction by the organizer-secreted factors chordin and noggin in *Xenopus* animal caps.. EMBO J.

